# A High Sensitivity and Wide Dynamic Range Fiber-Optic Sensor for Low-Concentration VOC Gas Detection

**DOI:** 10.3390/s141223321

**Published:** 2014-12-05

**Authors:** Md. Rajibur Rahaman Khan, Shin-Won Kang

**Affiliations:** School of Electronics Engineering, Kyungpook National University, 1370 Sankyuk-dong, Bukgu, Daegu 702-701, Korea; E-Mail: rajibur@ee.knu.ac.kr

**Keywords:** fiber-optic sensor, frequency modulation, volatile organic compound, solvatochromism, side-polished optical fiber

## Abstract

In this paper, we propose a volatile organic compound (VOC) gas sensing system with high sensitivity and a wide dynamic range that is based on the principle of the heterodyne frequency modulation method. According to this method, the time period of the sensing signal shift when Nile Red containing a VOC-sensitive membrane of a fiber-optic sensing element comes into contact with a VOC. This sensing membrane produces strong, fast and reversible signals when exposed to VOC gases. The response and recovery times of the proposed sensing system were less than 35 s, and good reproducibility and accuracy were obtained.

## 1. Introduction

Volatile organic compounds (VOCs) are common air pollutants found as ingredients in many household products. There are thousands of different VOCs used in our daily lives, and we may not be aware how or when VOCs are affecting our air quality. The definition of VOCs has been proposed by well-known organizations, including the World Health Organization (WHO) 1989 [1], the American Society for Testing and Material (ASTM) D3960-98 USA [2], the International Organization for Standardization (ISO) [3], the European Community [4], and the German Institute for Standardization (DIN) 55649-200, Germany [5]. According to their definition, VOCs are a group of chemical compounds that evaporate easily at room temperature. Along with carbon, they may contain other elements such as hydrogen, oxygen, fluorine, chlorine, bromine, sulfur, or nitrogen [6]. Concentrations of many VOCs are consistently higher indoors (up to ten times) than outdoors [7], and exposure to VOCs in the air has been linked to a number of short- and long-term adverse health effects, including cancer, liver damage, and kidney damage. The health risks from inhaling any chemical depends on its concentration in the air and on the exposure time and breathing rate of the people inhaling it. VOCs combined with nitrogen oxides react to form ground-level ozone, or smog, which contributes to climate change [6]. Therefore, the importance of detecting the presence of VOCs in indoor air goes beyond health concerns.

Over the last few decades, many sensors have been designed to detect VOCs, such as surface acoustic wave sensors [8,9], polymer composite sensors [10–12] carbon nanotubes [13–15], CMOS [16], and capacitive sensors [17]. Information about VOCs may be provided by colorimetric sensor array technology. The application of the colorimetric [18–20] sensor array is well known because those sensors are low cost, easy to fabricate, not dependent on a light source or subsidiary circuits, and can monitor different VOC gases in real time, but their main problem is that that they cannot detect the concentration of VOCs. Localized surface plasmon resonance (LSPR) sensors have become popular for VOC gas detection for their good sensing ability [21–24]. However, these sensors are expensive, have a complex fabrication process and are bulky.

Recently, fiber-optic sensors have been shown to be excellent candidates for sensor applications [25]. A side-polished fiber-optic sensor uses a combination of fiber-optic and optical waveguide technology to increase the sensor sensitivity to chemical, physical, or environmental changes [26–30]. These sensor systems lack the ability to detect a minuscule change in the light and are bulky. Yuan *et al.* [31] and Yeom *et al.* [32] developed a side-polished fiber-optic VOC sensor that was based on the principle of wavelength shift as the sensing membrane interacted with VOCs. This sensing principle is not new, and the main advantages of those types of sensors are known to be low cost, easy fabrication, and high sensitivity. However, these sensors have a low dynamic range, are difficult to obtain a resonance wavelength for, have high response and recovery times, and have unstable sensing performance.

In our study, we propose a low-cost, highly sensitive, easy-to-fabricate, fiber-optic VOC gas sensing system with wide dynamic range that is based on the principle of the optical heterodyne frequency modulation technique. According to this technique, the time period of the received sensing signal shift with respect to the reference signal time period, but the pulse width remains constant when the VOC sensitive optical sensing element comes into contact with the VOC.

The designed optical VOC gas sensing element was prepared by incorporating Nile Red, which has solvatochormic properties, into polyvinylpyrrolidone (PVP). It is then used as a sensing membrane on a side-polished single-mode fiber. When the sensing membrane comes into contact with the VOC gas, the refractive index of the polymer planar waveguide (PWG) causes changes to occur. This is due to the charge transfer (CT) bands of the solvatochromic dye in the VOC sensing element. Therefore, the small change in the refractive index of the PWG is shown as the specific shift of the received sensing signal with respect to the reference signal. For a side-polished fiber-optic sensor based on the amplitude modulation method cannot detect a very small change in the light. However, for the proposed sensing system, we observe the shift in optical signal which depends on the some parameters. As a result, the proposed fiber-optic VOC gas sensing system offers a linear sensing performance over a wide dynamic range, and can effectively detect low concentrations of VOCs.

We have developed highly sensitive side-polished fiber-optic sensor [33] and a multi-sensor array [34] VOC sensing systems based on the principle of the pulse width modulation technique (PWM), according to which the time period of the received sensing signal remains constant, but the pulse width of the received sensing signal changes as the concentration of the VOCs is changed, whereas in the proposed heterodyne frequency modulation sensing system the pulse width of the received sensing signal remains constant, but the time period of the received sensing signal shifts according to the concentration of VOCs gas. In addition, in the case of wavelength shift VOC sensing system [31,32] the resonance wavelength shift as the concentration of VOC is changed. Table 1 shows the differences among the VOC detection technique of the three different sensing systems.

The deposition process of the sensing membrane on the side-polished optical fiber block to make the optical sensing element of the proposed heterodyne frequency modulation sensing system and the wavelength shift VOC sensing system is approximately same, but there are some differences between them concerning the thickness of the polished cladding and sensing membrane. For example, the thickness of the sensing membrane of the proposed sensing system is thinner than in the wavelength shift VOC sensing system. Though the fabrication processes of the optical sensing element of the above sensing systems have similarities, the VOC detection principle of the proposed heterodyne frequency modulation sensing system is completely different than that of the PWM as well as the wavelength shift VOC sensing system. In addition, the heterodyne frequency modulation VOC sensing system offers a wider dynamic range than the PWM and wavelength shift VOC sensing systems.

Five different kinds of VOCs, namely dimethylamine, ethanol, benzene, toluene, and acetic acid, at concentrations from 0 to 5 ppm (5000 ppb) were used to observe the sensing ability of the proposed sensing system. The proposed heterodyne VOC gas sensing system was compared with the wavelength shift [31,32] test of a VOC sensing system and an LBJT [35,36] VOC sensor with respect to sensitivity, dynamic range width, linearity, and response and recovery times. We found that the proposed heterodyne frequency modulation VOC gas sensing system has better performance. The proposed fiber-optic VOC gas sensing system has several other advantages, such as real-time monitoring capabilities, good reproducibility, a linear sensing response over a large dynamic range, remote sensing capabilities, compactness, and low cost, as the circuitry is based on easily available and inexpensive opto-electronic components.

## 2. Theory and Operation Principle

Evanescent field coupling between a side-polished fiber and a dielectric waveguide overlay has been extensively studied for almost two decades. To create such an evanescent field fiber device, we have to remove a small part of the cladding layer in order to influence the intensity of the fields propagating in the fiber. The core cannot be damaged, and the removed part with quite a close distance to the core will be helpful to create high-performance devices. Several popular fiber-optic-based evanescent field sensor architectures are available, such as a de-clad cylindrical core, U-shaped de-clad cylindrical core, side-polished fiber block, and D-fiber [37–41]. In 1992, Tseng and Chen proposed a side-polished fiber block architecture with a single-mode fiber [26]. The key characteristics of this architecture, such as low cost, easy fabrication, low response time, and sensitivity, can be controlled by changing the bending radius of the polished fiber region as well as by reducing the thickness of the residual cladding. Chiu *et al.*, proposed a D-fiber architecture for an evanescent field absorption sensor [40]. This architecture is highly sensitive to chemical and biological sensing applications. However, this evanescent field fiber-optic architecture is expensive, has a complex fabrication process, and has a response time greater than the side-polished fiber block architecture. In our experiment, we used the side-polished fiber block architecture to create the optical sensing element.

Therefore, after deposition of the sensing membrane on the side-polished fiber block, there were two optical waveguides formed. When light propagates in an optical fiber, a fraction of the radiation extends for a short distance from the guiding region into the medium of a lower refractive index that surrounds it. This is the evanescent field and can be represented in the following form [42]:

(1) $$E\left(z\right)=E_{0}\exp\left(-\frac{z}{d_{p}}\right)$$

where *d_p_* is the perpendicular distance from the core-cladding interface at which the electric field amplitude has decreased to 1/*e* of the initial amplitude *E*_0_. It is called the penetration depth and is defined by the following mathematical equation [43]:

(2) $$d_{p}=\frac{\lambda }{2\pi n_{1}}{\left\{\sin^{2}\theta -{\left(\frac{n_{2}}{n_{1}}\right)}^{2}\right\}}^{-0.5}$$

where *n_1_* and *n_2_* are the refractive indexes of the fiber cladding and the material in contact with the top surface of the overlay, respectively. *λ* is the wavelength of the transmitted light, and *θ* is the angle of incidence normal to the interface. The sensitivity of the sensor depends on the penetration depth *d_p_*. The evanescent field energy may interact with analytes that attenuate it by means of refractive index changes, absorption, or scattering. The transmitted power of the light that passes through the optical fiber is dependent on the absorption of the evanescent field penetrating into the fluid in the polished cladding region. The transmitted power through the optical fiber with the cladding locally replaced by an absorbing medium is given by [44]:

(3) $$P_{L}=P_{0}\exp\left(-\gamma CL\right)$$

where *P_L_* is the power transmitted through the fiber with the absorbing medium over an polished-clad portion of length *L, P_0_* is the power transmitted through the fiber without an absorbing medium, *C* is the concentration of the absorbing medium, and *γ* is the evanescent wave absorption coefficient.

When the light pulse propagates through the fiber-optic-based waveguide, then the phase *φ* introduced by a field *E* over a polished fiber length *L* is given by [33,45]:

(4) $$\phi =\frac{2\pi n}{\lambda \gamma C}{\left(\frac{T}{T_{H}}\right)}^{2}$$

where *n* is the effective refractive index of the sensing membrane. *T* and *T_H_* are the time period and pulse width of the received sensing signal, respectively. From the derived mathematical term in Equation (4), it is easy to understand that the phase *ϕ* is a function of *λ,n, γ, C, T_H_*:

(5) $$\phi =f\left(\lambda ,n,\gamma ,C,T_{H},T\right).$$

Thus, the concentration of a VOC gas can be determined by observing the phase shift between the sensing and the reference signals, and the phase shift of the received signal increases when the concentration of gas increases and *vice versa*.

## 3. Experimental Details

### 3.1. Fabrication of the Side-Polished Optical Fiber Device

In our proposed sensing system, we used an optical VOC sensing element, which was made of a side-polished optical fiber device and a polymer planar waveguide deposited on it. The optical fiber is too small to be polished alone, so means to support the fiber during polishing are required. In our study we used a quartz block to support the optical fiber. The choice of quartz block is understandable because it has the same basic material as silica fiber. The fabrication process of the side-polished fiber-optic device is described in [33]. The bending radius of the optical fiber inside the quartz block was approximately 60 cm, and the length of the fiber was 1 m. Epoxy was first applied to the central section of the fiber. The fiber sections in the outer regions were glued after the epoxy in the central region was dry and hard. Then, the fibers were polished using l000 μm and 8000 μm polishing powders on polishing pads consecutively.

**Figure 1.**
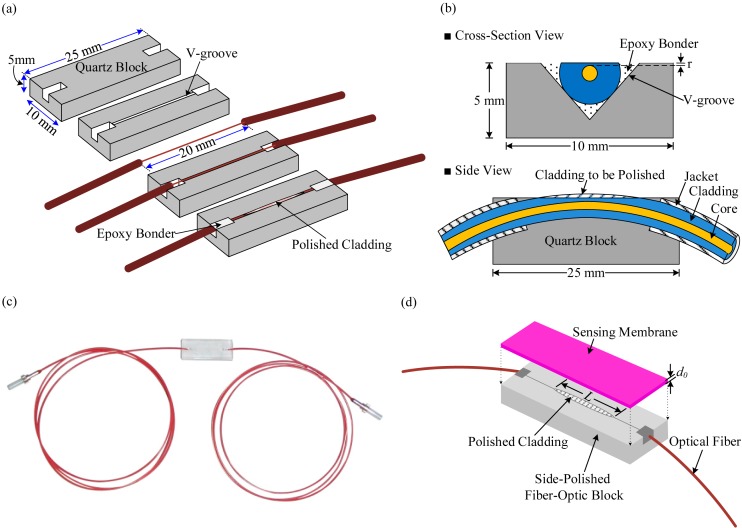
Side-polished optical fiber device: (**a**) step-by-step fabrication process; (**b**) schematic diagram of the cross-sectional view and side view of a fiber embedded in a quartz block (**c**) photograph of the manufactured side-polished fiber-optic block; and (**d**) schematic diagram of the side-polished optical fiber with a sensing membrane.

Figure 1a illustrates the step-by-step fabrication procedure of a side-polished fiber-optic block. A cross-sectional view of a fiber embedded in a V-groove is shown in Figure 1b. The distance *r* from the polished surface to the core is an important parameter determining the strength of interaction of a side-polished fiber with an external medium. The thickness of the residual cladding *r, i.e.*, the polished cladding thickness, was approximately 0.6 μm and was monitored using a drop method [26]. An image of the manufactured side-polished fiber block is shown in Figure 1c. Figure 1d shows the schematic diagram of a side-polished optical fiber with a sensing membrane.

### 3.2. Fabrication of the Sensing Membrane

The phenomenon whereby a compound changes color, either by a change in the absorption or emission spectra of the molecule, when dissolved in different solvents is called solvatochromism and is attributed to the difference in the ground state and excited state dipole moments [46]. Conceptually, when the ground state has a larger dipole moment, it will be preferentially stabilized by a more polar solvent, which will increase the transition energy gap and induce a hypsochromic shift in the spectrum, referred to as negative solvatochromism. Conversely, when the excited state has a larger dipole moment, it will be stabilized by a more polar solvent, and the transition energy decreases, which is observed as a bathochromic shift in the spectrum and is referred to as positive solvatochromism [46–48]. Solvatochromism has both optical and electrical characteristics. Nile Red has positive solvatochromic properties. Therefore, the solvent sensitivity of Nile Red is due to a significant increase in the dipole moment from the ground state to an excited state, which is a consequence of a large charge transfer between the donor (diethyl amino) and acceptor (carbonyl oxygen) moieties of Nile Red. Therefore, when the solvent polarity increases, then the energy band gap between the ground and the first excited state is reduced, and as a result, the mobility of charge increases, and a bathochromic (red) shift occurs. Because of the change of the energy band gap between the ground state and the first excited state, the relative permittivity of the molecule and the refractive index of the dye molecule change. The relationship between the relative permittivity and the refractive index can be written in the following mathematical form [49]:

(6) $$n=\sqrt{{ɛ}_{r}}$$

**Figure 2.**
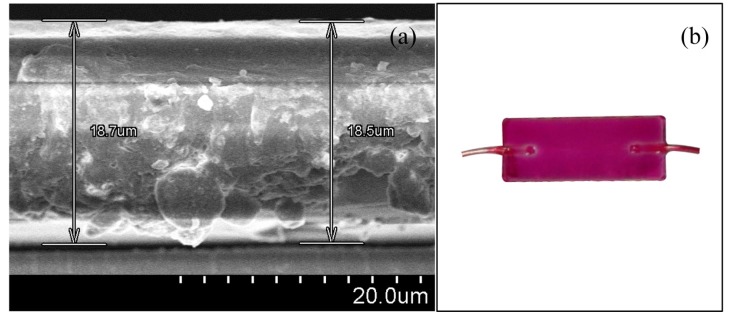
VOC sensitive sensing membrane: (**a**) SEM image of the thickness of the VOC sensing membrane and (**b**) photograph of the fabricated side-polished fiber-optic device after incorporation of the Nile Red-containing sensing membrane.

In our experiment, Nile Red dye, N,N-dimethylacetamide (DMAC, 99%), and PVP were used to prepare the sensing solution. All reagents were purchased from Sigma-Aldrich Chemical Corporation (St. Louis, MI, USA) and used without further purification. First, 0.55 wt% Nile Red and 50 wt% PVP were dissolved in 2 mL DMAC at a 1:1 volume ratio. The mixture was sonicated for 5 min to obtain the sensing solution. The side-polished fiber block was washed with acetone, methanol, and distilled water and dried with N_2_ gas. Then, the sensing solution was deposited on the side-polished portion of the fiber-optic block by spin-coating with three stages of spinning; 500 rpm for 5 s, 1000 rpm for 5 s, and 1500 rpm for 20 s. After deposition of the sensing solution, the fiber block was dried at room temperature. A scanning electron microscope (S-4800, Hitachi, Ibaraki, Japan) was used to measure the thickness of the VOC sensing membrane, which was approximately 18.6 μm. The SEM image of the thickness of the polymer PWG is shown in Figure 2a. The fabricated fiber-optic device after a sensing membrane was deposited on it is shown in Figure 2b.

### 3.3. VOC Detection System

**Figure 3.**
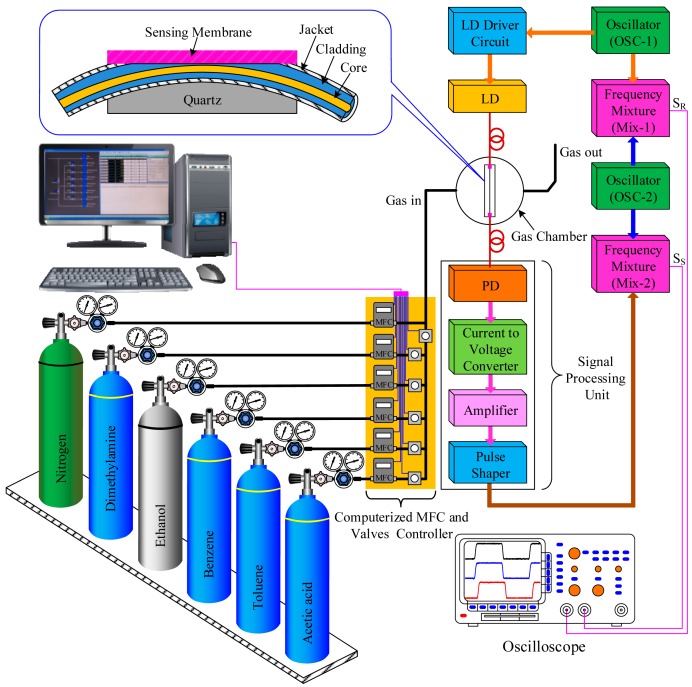
Schematic diagram of the experimental setup of the proposed heterodyne frequency modulation VOC sensing system.

A schematic diagram of the proposed heterodyne frequency modulation sensing system for the characterization of VOC gases is shown in Figure 3. It consists of a flow control system, gas tanks (VOCs and N_2_), test gas chamber, a side-polished optical fiber VOC sensing element, two oscillators (OSC-1 and OSC-2), two frequency mixers (Mix-1 and Mix-2), a laser diode (LD) (850 nm), a laser diode driver, a signal processing unit, and an oscilloscope (TDS3032B, Tektronix, Wilsonville, OR, USA). The signal processing unit is mainly divided into four parts: a light detector (PD), a current-to-voltage converter, an amplifier, and a pulse-shaping circuit. We have designed all electronic circuits of the VOC sensing system with easily available and inexpensive opto-electronic components.

In our experiment, the 850-nm laser light was modulated using a square wave by oscillator OSC-1 with a frequency 200 kHz. This was coupled to one end of the fiber-optic sensing element, and a light pulse was propagated along the fiber-optic sensing element. After traveling through the side-polished fiber-optic sensing element, the light from the other end of the fiber-optic sensing element was converted to an electrical signal by a photo diode (PD), and its output was connected to an operational amplifier in the current follower configuration. The output of the current follower was fed to the input of an amplifier for sufficient amplification. Then, the output of the amplified signal was inserted into the input of the pulse-shaping circuit. The output signal of the pulse shaping circuit was mixed with an electric signal from OSC-2, which had a frequency of 350 kHz by Mix-2. The mixed frequency is used as a measured heterodyne frequency signal S_S_. Simultaneously, another electrical signal from Mix-1, which is mixed by the frequency from OSC-1 and OSC-2, is used as a reference heterodyne frequency signal S_R_.

In our experiment, five different types of VOCs, namely dimethylamine, ethanol, benzene, toluene, and acetic acid, were used to observe the sensing performance of the proposed sensing system. The VOC gases was mixed with N_2_ gas (99.999%) to obtain the desired VOC concentrations. The concentration of the VOC gas was controlled using a computerized mass flow controller (MFC). The inner radius of the gas chamber was approximately 80 mm, and the height was approximately 75 mm. The side-polished fiber-optic sensing element was fixed inside the test gas chamber by chemically inactive double-sided tape. For sensor calibration, when no VOC gas is present in the gas chamber, the system is adjusted so that there is no phase shift difference. To test the response of the sensor, N_2_ gas is first injected into the test gas chamber and creates a stable baseline. Then, the gas under investigation is allowed to flow into the chamber, and a baseline is determined. Once the Nile Red-containing membrane is exposed to the VOC gas, the refractive index of the PWG changes. This causes a shift of the optical phase of the sensing signal. The phase shift difference between the sensing and reference signals is measured at the room temperature and recorded using an oscilloscope.

## 4. Results and Discussion

**Figure 4.**
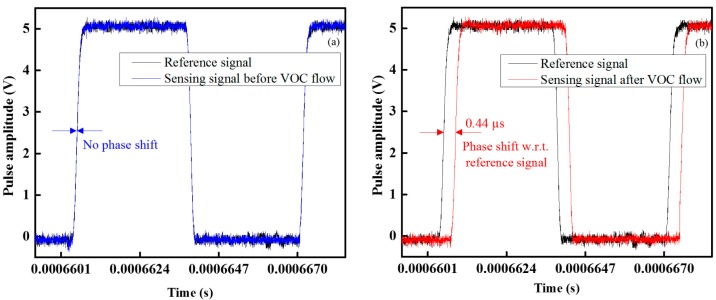
Waveform response: (**a**) sensing and reference signal and (**b**) sensing signal shift with respect to reference signal.

The waveforms of the sensing and reference signals under ideal conditions with no VOC gas in the gas chamber are shown in Figure 4a; there was no phase difference of the sensing signal with respect to the reference signal. On the other hand, when VOC gas flowed into the gas chamber, the sensing signal shift with respect to the reference signal and was measured by an oscilloscope (Tektronix TDS3032B), as shown in Figure 4b. From Figure 4b, it is seen that the time period of the sensing signal shift with respect to the reference signal time period at the benzene gas concentration of 3 ppm was 0.44 μs and the pulse width remain constant, while in the case of PWM sensing system in [33] the time period of the sensing signal does not shift but the pulse width of the received sensing signal change according to the concentration of VOC gas.

**Figure 5.**
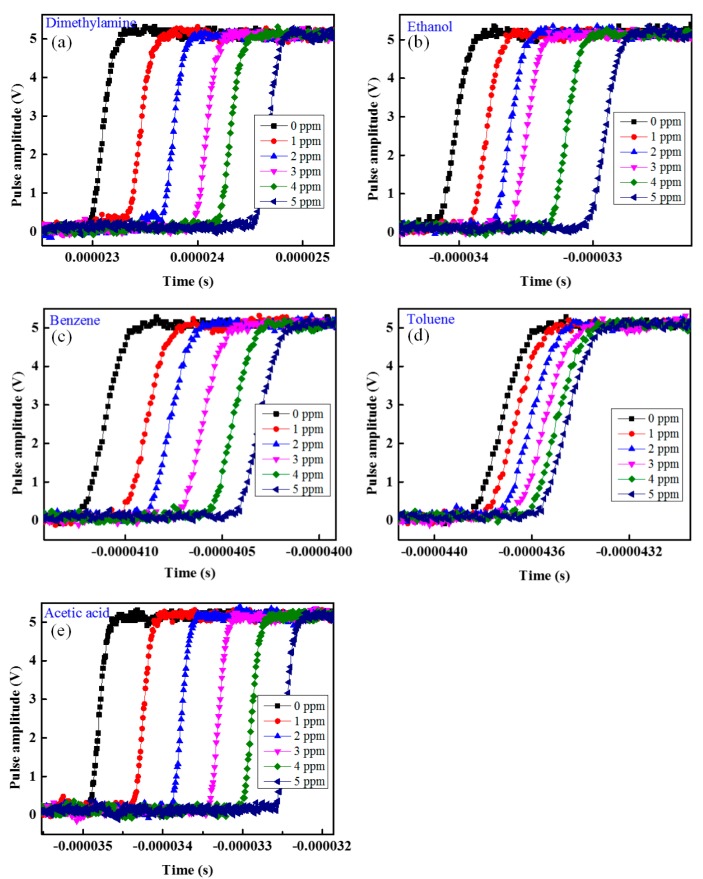
Phase shift change as the concentration of VOC change: (**a**) dimethylamine; (**b**) ethanol; (**c**) benzene; (**d**) toluene and (**e**) acetic acid.

To observe the VOC sensing performance of the proposed sensing system with respect to different VOCs and concentrations, we injected different VOCs, namely dimethylamine, ethanol, benzene, toluene, and acetic acid, individually into the test gas chamber. All measurements were taken at room temperature. Figure 5 shows the change in phase shift with respect to different VOCs and concentration from 0 to 5 ppm in increments of 1 ppm of dimethylamine, ethanol, benzene, toluene, and acetic acid, respectively. The response of a sensor is the phase shift difference between the sensing signal and the reference signal. From the experimental results in Figure 5 it is found that the as the concentration of VOCs increases then the phase difference between the reference and sensing signal also increases and *vice versa*.

**Figure 6.**
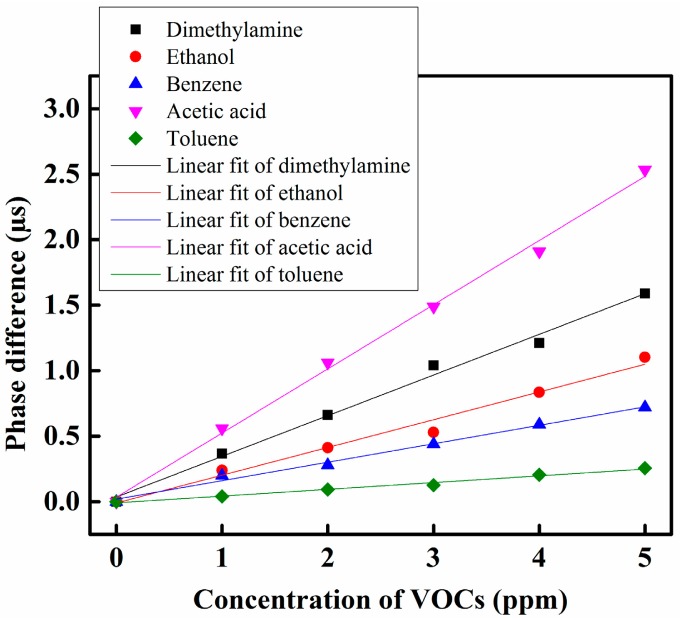
VOC response of the proposed sensing system with different VOCs.

The VOC response curves of the proposed sensing system under different VOCs are shown in Figure 6. According to the experimental observation, the proposed VOC gas sensing system offers a linear sensing performance over the dynamic range from 0 to 5 ppm. Most of the side-polished optical fiber VOC gas sensing systems [32–34] offered a dynamic range under 100 ppb, but the proposed heterodyne frequency modulation VOC gas sensing system offered a dynamic range 0 to 5000 ppb (5 ppm), which was wider in comparison with the other side-polished fiber-optic VOC gas sensing systems. Hence it can be concluded that the proposed sensing system has a wide dynamic range. From the sensing response of the sensor system, it can be observed that the lowest detections correspond to toluene gas, and the highest detections correspond to acetic acid gas. Selectivity is one of the characteristics of a gas sensor to recognize the presence of the particular gases in media including other gases. In our proposed heterodyne frequency modulation VOC gas sensing system we used five different types of VOC gases to observe the sensing performance. Those VOCs gases have different functional groups. The functional groups of dimethylamine, ethanol, benzene, toluene, and acetic acid are amine, alcohol, aromatic, aromatic, and carboxylic acid, respectively. According to the solvent polarity chart the carboxylic acid group is more polar than amines, alcohols, and aromatic groups. Moreover, dimethylamine, ethanol and acetic acid are polar gases while benzene and toluene are the non-polar gasesd. We used a Nile Red-containing sensing membrane to make the optical sensing element. Nile Red has a positive solvatochromic property. Therefore, when a polar gas such as acetic acid comes into the contact with the Nile Red sensing membrane, then the refractive index of the Nile Red-containing sensing membrane increases more than with a non-polar gas such as toluene. As a result, the phase shift of the received sensing signal in the case of acetic acid increases more than for toluene gas. There are some solvatochromic dyes which show a higher refractive index changes for a particular VOC gas for the same concentration of gas, thus, Nile Red shows a higher refractive index change in acetic acid gas. Similarly, Reichardt's dye (R-dye) and 4-amino-N-methylphthalimide (4-ANMP) dyes shows higher refractive index changes for dimethylamine and toluene gas, respectively [34]. In our proposed heterodyne frequency modulation VOC sensing system we used only a Nile Red-containing optical sensing element. Therefore, if we flow two different types of VOC gases such as acetic acid and benzene together in the gas chamber, then the response curve of the mixed VOCs gas will take place near the acetic acid curve in Figure 6 because acetic acid is more polar than benzene. To distinguish different VOC gases within a mixed VOCs gas we have to use an array sensing system. We have future plans to make a heterodyne frequency modulation array sensing system to distinguish different VOC gases within a VOC gas mixture.

**Figure 7.**
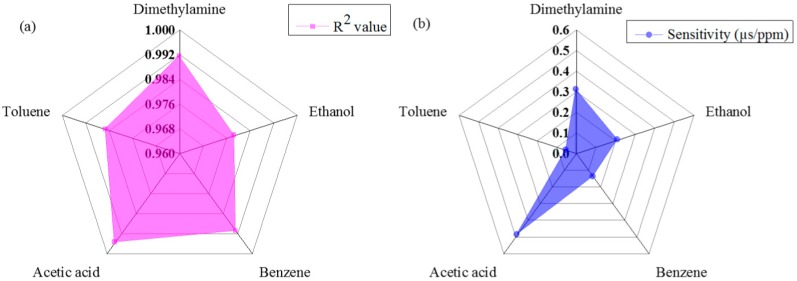
Performance of the proposed sensing system for different VOCs: (**a**) linearity and (**b**) sensitivity.

The linearity performance of the proposed sensing system under different VOCs using s radar chart is shown in Figure 7a. According to the results, it was found that the proposed sensing system offers the highest linearity with acetic acid gas, with an R^2^ value of approximately 0.9957, and the sensing system offered the lowest linearity performance with ethanol gas, with an R^2^ value of approximately 0.9787 over the dynamic range.

The sensitivity of the proposed sensing system under different VOCs using the radar chart is shown in Figure 7b. According to the results, we have found that the proposed sensing system shows higher sensitivity for acetic acid gas and shows lower sensitivity for toluene among the five different VOC gases tested. The sensitivity of the proposed sensing system for acetic acid and toluene gas was approximately 0.49 μs/ppm and 0.05 μs/ppm, respectively.

**Figure 8.**
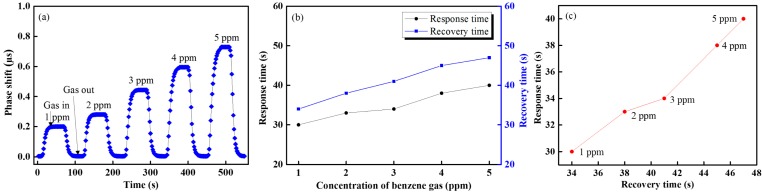
Heterodyne frequency modulation VOC gas sensor performance of the (**a**) real-time gas sensing responses of benzene (**b**) response and recovery times and (**c**) response *versus* recovery times of different concentration of benzene gas.

Figure 8 shows the response and recovery times for the proposed VOC sensing system and it is found that our proposed VOC gas sensing system has a shorter 30 s response time and 35 s recovery time. It also was determined from Figure 8b that the response and the recovery times were approximately proportional to the increase in the gas concentration. Figure 8c shows the response *versus* recovery times of benzene gas of concentration from 1 to 5 ppm. The proposed VOC sensing system offers a stable sensing performance over the dynamic range.

We were able to test the performance of our proposed sensing system with different sensing systems. One of them was based on the principle of wavelength shift [31,32], and another was based on the gated lateral bipolar junction transistor (LBJT) [35,36] VOC sensing system. We found that the proposed sensing system offers a large dynamic range, more linear sensing performance (correlation coefficient of R^2^ = 0.9884, approximately), short response and recovery time, and better sensing stability in comparison with the wavelength shift method as well as the LBJT VOC sensing system. For example, the response and the recovery times in [31] were less than 60 s, and in [36] response time and recovery times was 480 s and 450 s, respectively, whereas in our proposed sensing system, the response and the recovery times were found to be less than approximately 35 s. Moreover, the slope, *i.e.*, sensitivity of the proposed sensing system, was higher than the wavelength shift method and LBJT VOC sensing system.

## 5. Conclusions

In this study we have designed and developed a new type of highly sensitive VOC gas sensing system with a wide dynamic range using the evanescent field coupling between a side-polished optical fiber and the polymer planar waveguide. The sensing principle is based on the heterodyne frequency modulation technique. According to this technique, the time period of the sensing signal shifts with respect to the reference signal when the VOC sensing membrane of the sensing system comes into contact with the VOC. Nile Red, whose optical characteristics change as it reacts with VOCs, was incorporated into polyvinylpyrrolidone (PVP) and fabricated into a sensing membrane by a spin coater on a side-polished single-mode fiber to prepare the fiber-optic sensing element of the sensing system. Five different kinds of VOCs, namely dimethylamine, ethanol, benzene, toluene, and acetic acid, were used to observe the performance of the sensing system. The proposed VOC gas sensing system has many advantages, including easy fabrication, a linear response over a dynamic range, a short response time, remote sensing capabilities, compactness, and low cost, as the circuitry is based on easily available and inexpensive opto-electronic components. According to the results, the dynamic range of VOCs varied from 0 ppm to 5 ppm. The response properties of the proposed VOC gas sensing system were linear and had good reproducibility. We expect that any sensor device produced using the same fabrication process under the same conditions would show good stability. In future studies, other sensing materials will be used in the system to detect different VOC gases. We also plan further experiments on gas device arrays and multi-sensing based on the results of this experiment.

## Figures and Tables

**Table 1.** Detection technique of the three different sensing systems: Proposed sensing system, PWM sensing system, and wavelength shift sensing system.

	**Detection Technique**		
	Proposed sensing system	PWM sensing system	Wavelength shift sensing system
Waveform Analysis	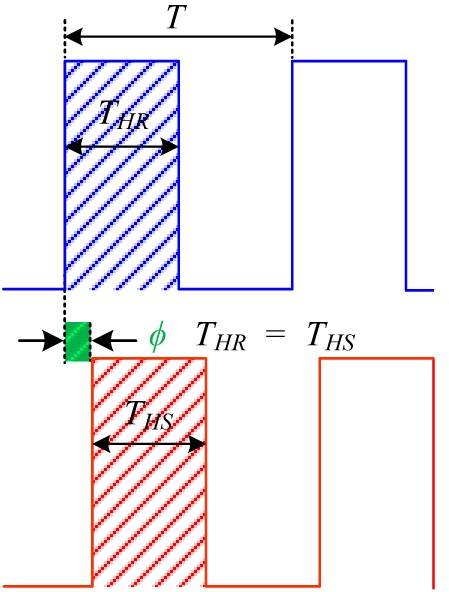	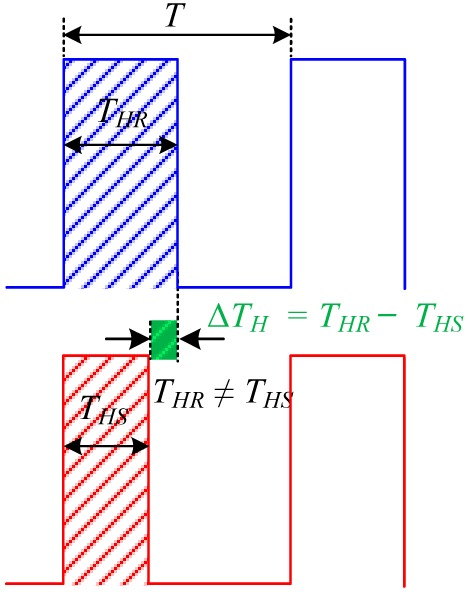	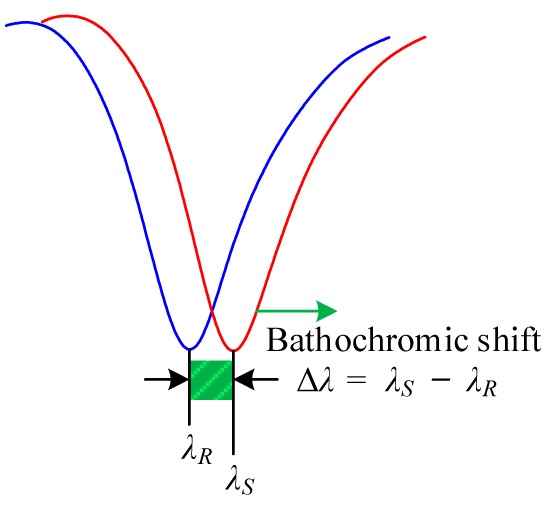
Response	Time period *T* shift Pulse width remain fixed *t_HR_* = *t_HS_*	Time period *T* remain fixed Pulse width change Δ*T_H_* = *T_HR_* − *T_HS_*	Resonance wavelength shift with respect to reference wavelength Δ*λ* = *λ_S_* − *λ_R_*
Notation	*T* = Time period of signal *T_HR_* = Pulse width of the reference signal *T_HS_* = Pulse width of the sensing signal Δ*T_H_* = *T_HR_* − *T_HS_* = Pulse width difference *ϕ* = Phase shift between the reference and the sensing signal *λ_R_* = Resonance wavelength before VOC flow *λ_S_* = Resonance wavelength after VOC flow Δ*λ* = − *λ_S_λ_R_* = Resonance wavelength shift		
